# Epstein-Barr virus-driven lymphomagenesis in the context of human immunodeficiency virus type 1 infection

**DOI:** 10.3389/fmicb.2013.00311

**Published:** 2013-10-18

**Authors:** Maria R. Petrara, Riccardo Freguja, Ketty Gianesin, Marisa Zanchetta, Anita De Rossi

**Affiliations:** ^1^Viral Oncology Unit and AIDS Reference Center, Section of Oncology and Immunology, Department of Surgery, Oncology and Gastroenterology, University of PadovaPadova, Italy; ^2^Istituto Oncologico Veneto - Istituto di Ricovero e Cura a Carattere ScientificoPadova, Italy

**Keywords:** EBV, HIV-1, B cell activation, chronic immune activation, EBV-related malignancies, antiretroviral therapy, EBV lytic reactivation

## Abstract

Epstein–Barr virus (EBV) is a ubiquitous human γ-herpes virus which establishes a life-long asymptomatic infection in immunocompetent hosts. In human immunodeficiency virus type 1 (HIV-1) infected patients, the impaired immunosurveillance against EBV may favor the development of EBV-related diseases, ranging from lymphoproliferative disorders to B cell non-Hodgkin’s lymphomas (NHL). Antiretroviral therapy (ART) has significantly modified the natural course of HIV-1 infection, resulting in decreased HIV-1 plasmaviremia, increased CD4 lymphocytes, and decreased opportunistic infections, indicating a restoration of immune functions. However, the impact of ART appears to be less favorable on EBV-related malignancies than on other AIDS-defining tumors, such as Kaposi’s sarcoma, and NHL remains the most common cancer during the ART era. EBV-driven tumors are associated with selective expression of latent oncogenic proteins, but uncontrolled lytic cycle with virus replication and/or reactivation may favor cell transformation, at least in the early phases. Several host’s factors may promote EBV reactivation and replication; besides immunodepression, inflammation/chronic immune stimulation may play an important role. Microbial pathogen-associated molecular patterns and endogenous damage-associated molecular patterns, through Toll-like receptors, activate the immune system and may promote EBV reactivation and/or polyclonal expansion of EBV-infected cells. A body of evidence suggests that chronic immune stimulation is a hallmark of HIV-1 pathogenesis and may persist even in ART-treated patients. This review focuses on lymphomagenesis driven by EBV both in the context of the natural history of HIV-1 infection and in ART-treated patients. Understanding the mechanisms involved in the expansion of EBV-infected cells is a premise for the identification of prognostic markers of EBV-associated malignancies.

## INTRODUCTION

Epstein–Barr virus (EBV) is a ubiquitous human γ-herpes virus which establishes a life-long asymptomatic infection in immunocompetent hosts. Most individuals contract EBV infection by early adulthood; primary EBV infection is usually asymptomatic, but sometimes results in infectious mononucleosis, a self-limiting disease due to the EBV-specific immunological response. Regardless of its iatrogenic or infectious origin, immunodeficiency increases the risk of tumor development, particularly of tumors etiologically linked to viral agents. In post-transplant patients and in individuals infected with human immunodeficiency virus type 1 (HIV-1), impaired immunosurveillance against EBV may favor the development of EBV-associated diseases, ranging from lymphoproliferative disorders to B cell non-Hodgkin’s lymphomas (NHL; Ometto et al., 1997; Beral and Newton, 1998). Antiretroviral therapy (ART) partially restores immune functions and has significantly changed the course of HIV-1 infection (Palella et al., 1998). However, the impact of ART seems to be less favorable on EBV-related diseases than on other AIDS-defining events, including Kaposi’s sarcoma (KS; Simard et al., 2011). Notably, NHL has been considered as an AIDS-defining cancer (ADC) since 1985, and remains one of the main causes of death in HIV-1 infected patients even in the ART era [Collaboration of Observational HIV Epidemiological Research Europe (COHERE) Study Group, 2009; Simard et al., 2011].

## INCIDENCE OF EBV-RELATED MALIGNANCIES IN HIV-1 INFECTED PATIENTS

There are several subtypes of AIDS-associated NHL, including Burkitt’s lymphoma (BL), immunoblastic lymphoma (IBL), diffuse large B cell lymphoma (DLBCL), and primary central nervous system lymphoma (PCNSL). The frequency of EBV detection in these tumors ranges from 60% in BL to 100% in PCNSL (Carbone et al., 2009). Before the introduction of ART, the incidence of NHL in HIV-1 infected subjects was 100 times higher than in the general population (Goedert et al., 1998; International Collaboration on HIV and Cancer, 2000), PCNSL and high grade IBL being the most common NHL subtypes, followed by BL and intermediate grade DLBCL (Kaplan, 2012).

High levels of HIV-1 plasmaviremia and low CD4 cell counts are both risk factors for the onset of NHL. The risk substantially increases in patients with >100,000 HIV-1 RNA copies/ml and/or with <50 CD4 cells/μl (Engels et al., 2010). However, systemic lymphomas may occur at virtually any level of CD4 cell count, BL occurring more frequently in patients with moderate/advanced immunodepression (200–500 CD4 cells/μl) and PCNSL occurring in patients with severe immunodepression (<200 CD4 cells/μl).

The introduction of ART into clinical practice has modified the natural course of HIV-1 infection, resulting in the decrease of HIV-1 plasmaviremia, increase in CD4 T lymphocytes, and decrease in HIV-1-associated opportunistic infections. Deaths from AIDS-related diseases have fallen by 75% (Tirelli and Bernardi, 2001) and people diagnosed with HIV-1 can expect to live for 30 or 40 years after contracting the infection, equivalent to a reduction in lifespan of about one-third (Grulich, 2009). Therefore, patients with HIV-1 infection are now living longer, with improved immune functions and reduced risk of developing AIDS; nevertheless, ADCs remain higher when compared with the rate of these cancers in the general population (Petoumenos et al., 2013).

After the introduction of ART, the overall incidence of NHL declined by nearly 50% compared with ART-untreated patients (International Collaboration on HIV and Cancer, 2000; Biggar et al., 2007; Simard et al., 2011); the adjusted incidence rate of NHL was estimated to decline from 6.2% pre-ART (1992–1999) to 3.2% in the ART era (1997–1999; International Collaboration on HIV and Cancer, 2000). Five-year cumulative incidence of NHL declined from 3.8% during the 1990–1995 period to 2.2% during 1996–2006 (Simard et al., 2011). This pattern is quite different from that observed with KS, associated with human herpes virus 8 (HHV8; Cattelan et al., 1999; Simard et al., 2011). The adjusted incidence rate of KS declined from 14.3% pre-ART to 1.8% in the ART era (Simard et al., 2011). After the introduction of ART, NHL is still the most common ADC. In addition, the decline is not equally distributed among the various NHL subtypes, but is higher for PCNSL and lower for the other subtypes.

The increased risk of NHL cannot be explained by immunodepression alone. Patients treated with ART may survive longer, with continued B cell stimulation, leading to an increased incidence of lymphoma over time. Emerging data also indicate that HIV-1 infection, even among long-term ART-treated patients, is associated with a higher rate of non-AIDS-defining malignancies (NADC; Deeken et al., 2012); this evidence further supports the concept that immunodeficiency is not sufficient to explain the higher risk of cancer in HIV-1 infected subjects. The substantial reduction in HIV-1-associated mortality and the increase in an aging population with HIV-1 may contribute to the rise in NADC, including some tumors associated with co-infections [e.g., anal and oropharyngeal cancer associated with human papillomavirus, liver cancer associated with hepatitis B and C viruses, and Hodgkin’s lymphoma (HL) associated with EBV]. In a cohort of HIV-1 infected European patients, the incidence of HL was 4.5 times higher than in the general population pre-ART (1983–1985) and 32 times higher in the ART era (2002–2007; Powles et al., 2009). These results have been replicated in a Swiss HIV-1 cohort, in which the incidence of HL increased 9 times pre-ART, 21 times in the early ART era, and 28 times in the late ART era (Franceschi et al., 2010). Also, in a United States cohort, the 5-year cumulative incidence of HL increased from 0.09% pre-ART (1980–1989) to 0.19% in the ART era (1996–2006; Simard et al., 2011). HIV-1-associated HL differs from HL in HIV-1 uninfected patients, because it is almost always associated with EBV and more often presents systemic “B” symptoms, and extranodal involvement (Jacobson and Abramson, 2012).

## EBV-DRIVEN TUMORIGENESIS

Epstein–Barr virus is associated with both B cell and epithelial cell malignancies. Like all herpes viruses, EBV has both latent and lytic replication programs, allowing it to evade immune clearance and persist for the host’s lifetime. Latent infection is characterized by replication of the viral genome as an integral part of the host cell chromosomes and the absence of production of infectious virus. Since lytic EBV replication triggers the death of infected cells, tumors require the expression of latent programs. Latent proteins include nuclear antigens (EBNA-1, -2, -3A, -3B, and -3C), leader protein (LP), and latent membrane proteins (LMP-1, -2A, and -2B). LMP-1 is the main oncogenic protein of EBV and is essential for EBV-driven tumorigenesis. It is expressed in all NHL, except BL, and also in HL. Functionally, LMP-1 mimics CD40, a member of the tumor necrosis factor receptor (TNFR) superfamily, thus providing both growth and differentiation signals to B cells. LMP-1 does in fact activate several downstream signaling pathways which contribute to the induction of genes encoding anti-apoptotic proteins (e.g., BCL-2 and A20) and cytokines, such as interleukin (IL)-1, and CD40L (Young and Rickinson, 2004; Chen, 2011).

*In vitro*, EBV infection of B cells may generate immortalized lymphoblastoid B cell lines (LCL) which express all the latent viral genes, as most EBV-NHL do; this is why LCL are often employed as a model to study EBV-driven lymphomagenesis. Besides the expression of viral latent proteins, the transformation of primary B lymphocytes by EBV requires the activation of telomerase (Dolcetti and De Rossi, 2012). Telomerase is a ribonucleoprotein complex, containing an internal RNA, used as a template to elongate telomeres, and a catalytic protein with telomerase reverse transcriptase (TERT) activity. Telomerase is critically involved in maintaining telomere length and its activation is required for cells to overcome replicative senescence and acquire unlimited replicative potential, a prerequisite for neoplastic transformation (Blackburn et al., 2006). It has been demonstrated that LMP-1 activates *TERT* at transcriptional level *via* nuclear factor kappa B (NF-κB) and MAPK/ERK1/2 pathways (Terrin et al., 2007, 2008). In turn, TERT expression confers resistance to apoptotic stimuli and to the induction of the lytic cycle (Giunco et al., 2013).

Although latency programs predominate in EBV-driven tumors, recent evidence suggests that lytic EBV replication has some pathogenic importance, at least in the early phases of cell transformation. Several lytic proteins expressed during the lytic cycle of infection are involved in immune evasion. They include the *BCRF-1* gene which encodes a viral IL-10 cytokine which, like human IL-10, inhibits the synthesis of interferon-γ (IFN-γ) and suppresses CD8 cytotoxic T cell (Draborg et al., 2012). Recent studies also suggest that the lytic cycle contributes to the growth of EBV-associated malignancies by enhancing angiogenesis. Indeed, B cells infected with a virus competent for the expression of the lytic protein BZLF-1 released more vascular endothelial growth factor and IL-6 than cells infected with BZLF-1-defective virus (Hong et al., 2005). A new humanized mouse model, in which both human fetal CD34 hematopoietic stem cells and thymus/liver tissue were transplanted, was employed to test EBV-driven lymphomagenesis; mice injected with lytic replication-defective BZLF1-deleted virus developed fewer lymphomas than animals infected with lytic BZLF1-competent virus, suggesting that lytically infected cells promote EBV-driven lymphomagenesis (Ma et al., 2011).

## DIRECT AND INDIRECT INTERACTIONS OF HIV-1 WITH B CELLS AND EBV

Besides impaired immunosurveillance against EBV-infected cells due to the loss of T cell function, HIV-1 may contribute to the genesis of EBV-associated NHL through chronic B cell hyperactivation. Chronic systemic immune activation is a hallmark of HIV-1 pathogenesis; being one of the strongest predictors of disease progression (Deeks et al., 2004; Hunt et al., 2008) and it is also associated with impaired immune reconstitution during ART (Anselmi et al., 2007).

Immune activation involves both T cell and B cell compartments. In the former, immune activation may be assessed by the high frequency of T cells expressing markers of activation, such as CD38 and HLA-DR (Hazenberg et al., 2000; Anselmi et al., 2007), high levels of T cell proliferation (Hellerstein et al., 2003), and activation-induced apoptosis of HIV-1 uninfected T cells (Gougeon et al., 1996). In the B cell compartment, B cells express high levels of CD80 and CD86 co-stimulatory molecules. Several changes in the B cell subsets have also been described in association with HIV-1 plasmaviremia and/or CD4 cell decline. These changes include an increase in activated (CD27^+^CD21^low^) and exhausted (CD27^-^CD21^low^) B cells (Moir and Fauci, 2009), a decrease in circulating memory (CD27^+^) B cells (Titanji et al., 2006), and an increase in immature/transitional (CD27^-^CD10^+^) B cells (Malaspina et al., 2006). Immune activation may result in polyclonal B cell activation, hypergammaglobulinemia, increased cell turnover (Moir and Fauci, 2009), and ultimately increased frequency of B cell malignancies (Grulich et al., 2000).

The factors contributing to B cell hyperactivation and expansion of EBV-infected B cells remain largely unknown. The mechanisms responsible for polyclonal B cell activation seem to be multiple, and due to a combination of both direct and indirect interaction of B cells with HIV-1. HIV-1 itself can induce polyclonal B cell activation *in vitro* (Schnittman et al., 1986). It has been demonstrated that CD40L is incorporated into the HIV-1 envelope (Ambinder et al., 2010), and that B cells derived from the peripheral blood of HIV-1 infected viremic patients carry replication-competent virus on their membrane (Moir et al., 2000). There is evidence that HIV-1 binds to B cells through interactions between the complement receptor CD21, which is expressed on most mature B cells (Moir et al., 2000); the virus bound to B cells can efficiently infect CD4 T cells through cell–cell contact (Malaspina et al., 2002). A model of “self-limiting” HIV-1 infection of B cells has been described. After *in vitro* exposure to HIV-1, a steady-state fraction of LCL resulted positive for HIV-1 gp120 envelope protein. These cells underwent down-regulation of BCL-2 and death by apoptosis; the persistent fraction of gp120^+^ cells was maintained by HIV-1 transmission to B cells newly arising from the proliferation of gp120^-^ cells (De Rossi et al., 1994). It was also demonstrated that a fraction of B cells may bind gp120 through mannose C-type lectin receptors (MCLRs). In the presence of gp120, MCLR-expressing B cells up-regulate the activation-induced cytidine deaminase, and undergo immunoglobulin class switching (He et al., 2006). By contrast, HIV-1 protein Nef suppresses class switch recombination on B cells by interfering with CD40L-mediated signaling (Qiao et al., 2006). Nef also promotes polyclonal B cell activation and increases CD4 T cell susceptibility to HIV-1 infection by inducing macrophages to secrete pro-inflammatory cytokines (Swingler et al., 2008). Additional *in vitro* evidence of direct interaction between HIV-1 and several receptors on B cells have been reported (Berberian et al., 1993; Rappocciolo et al., 2006), however, to date there is still little evidence that HIV-1 can productively replicate in B cells *in vivo*.

The transactivator Tat protein of HIV-1 is released by HIV-1 infected cells and is taken up by surrounding HIV-1-uninfected cells through interactions with cell membrane heparan sulfate proteoglycans (Frankel and Pabo, 1988; Tyagi et al., 2001). HIV-1 binds to B cells which are in close contact with HIV-1-infected macrophages and to dendritic cells in the extranodal sites where NHL usually occur (Sparano, 2001). This cell–cell contact may favor B cell uptake of the protein; Tat protein has in fact been consistently detected in the neoplastic cells of NHL occurring in HIV-1 infected subjects (Lazzi et al., 2002). Tat modulates the expression of several cellular genes, including cytokines and their receptors, and increases the expression of IL-6 and IL-10 (Scala et al., 1994; Blazevic et al., 1996), which in turn promote B cell activation. It has been demonstrated that Tat prevents cell cycle arrest and confers resistance to apoptosis in EBV-infected B cells cultured *in vitro* in serum starvation conditions (Colombrino et al., 2004).

Chronic B cell hyperactivation is driven by overproduction of B cell stimulatory cytokines, such as IL-6, IL-10, interferon-α (IFN-α), and tumor necrosis factor (TNF; Rieckmann et al., 1991; Takeshita et al., 1995; Mandl et al., 2008). The serum levels of these B cell stimulatory cytokines and other molecules, such as soluble sCD27 and sCD30 generated from the corresponding B cell receptors during the process of immune activation, were significantly high 1–5 years prior to diagnosis of systemic AIDS-NHL (Ambinder et al., 2010; Breen et al., 2011). Elevated serum levels of IL-6 have also been observed 1–3 years prior to the onset of AIDS-NHL, thus supporting the role of IL-6-driven B cell stimulation in the development of these lymphomas (Breen et al., 2011).

Lastly, an important source of immune activation in HIV-1 infected individuals is provided by microbial translocation due to damage to intestinal mucosa caused by massive HIV-1-induced T cell depletion in the gut (Brenchley et al., 2006). Translocation of intestinal bacteria and bacterial products into the bloodstream can activate the immune system by binding to receptors involved in the host inflammatory response, such as Toll-like receptors (TLRs). TLRs are pattern recognition receptors which recognize structural components belonging to bacteria, fungi, and viruses, known as “pathogen-associated molecular patterns” (PAMPs), and activate the innate immune response (Janeway and Medzhitov, 2002). PAMPs include bacterial lipopolysaccharide (LPS), 16S ribosomal DNA (16S rDNA), and CpG DNA. A recent study demonstrated that levels of PAMPs, generated by microbial translocation (sCD14 and LPS) are associated with the risk of NHL (Marks et al., 2013). TLR9 substantially suppresses BZLF-1-mRNA expression by histone modifications in acute EBV infection *ex vivo* and in latently BL cells *in vitro*, suggesting that immune activation can also promote EBV-driven lymphomagenesis by suppressing the viral lytic cycle (Ladell et al., 2007). Patients who developed PCNSL expressed more CD80 and CD86 in their B cells and responded to TLR9 agonist better than patients without tumors (Audigé et al., 2010).

The loss of mucosal surface integrity in the gut, due to the massive depletion of CD4 T cells, involves not only increased mucosal permeability and consequent microbial translocation, but also an increase in “damage-associated molecular patterns” (DAMPs), endogenous molecules released after cell death, such as mitochondrial DNA (mtDNA; Zhang et al., 2010), high mobility group box 1 (HMGB1) protein (Scaffidi et al., 2002) and defensins (Sørensen et al., 2005). The binding of PAMP and DAMP ligands to the extra- or intra-cellular domain of TLRs initiates a complex-signal transduction cascade which, *via* the NF-κB pathway, ultimately leads to increased transcription of pro-inflammatory cytokines, such as IL-6 and TNF. During chronic infection, PAMPs and DAMPs may act in synergy on the TLR response. Thus, chronic inflammation may favor an environment which triggers tumor development.

## IMPACT OF ANTIRETROVIRAL THERAPY ON EBV LYMPHOMAGENESIS

The impact of ART is less favorable on EBV-related malignancies than on other ADC. In addition, the decline after the introduction of ART was more substantial in PCNSL than in other NHL subtypes. Because of the role played by chronic immune activation in the genesis of NHL, it may be argue that this mechanism (partially) persists even during ART. A study performed on a cohort of HIV-1-infected patients under ART disclosed a decline in both EBV plasmaviremia and citoviremia in patients with immunological (increase in CD4 cells) and virological (decline in HIV-1 plasmaviremia to undetectable levels) responses. Notably, a decrease in EBV load was also detected in patients with only virological response to ART. By contrast, the increase in CD4 cells without suppression of HIV-1 plasmaviremia was accompanied by an increase in EBV load, paralleled by an increase in immunoglobulin levels (Righetti et al., 2002). In agreement, EBV-DNA load was higher in patients with detectable HIV-1 plasmaviremia, despite good immunological status (>500 CD4 cells/μl) than in patients with undetectable HIV-1 plasmaviremia, regardless of immunological status (Petrara et al., 2012). It has been observed that ART only partially normalizes the serum cytokine levels; IL-6 remain at high levels even 2–3 years after ART initiation (Regidor et al., 2011). These findings strongly suggest that immune reconstitution without reduction in HIV-1 viremia may increase B cell stimulation and the number of EBV-infected cells; this may be important for new therapeutic strategies against HIV-1 to implement immune reconstitution and/or to alleviate adverse drug effects.

HIV-1 infected patients, treated with ART in combination with IL-2 to improve immunological reconstitution, showed a decrease in EBV plasmaviremia and citoviremia when treated with low-intermittent IL-2 doses, but presented an increase in EBV in both cells and plasma when treated with high doses of IL-2 (Burighel et al., 2006). Thus, according to dose, IL-2 may significantly affect EBV dynamics. Low doses of IL-2, by binding to high-affinity receptors expressed on T lymphocytes, may promote the activity of cytotoxic T lymphocytes, thus restoring protective immunity against EBV. Notably, low doses of IL-2 prevented the development of EBV-associated disease in severe combined immunodeficiency (SCID) mice reconstituted with peripheral blood cells from EBV-seropositive subjects (Baiocchi and Caligiuri, 1994). Conversely, high doses of IL-2, by binding low-affinity receptors expressed on natural killer (NK) cells, may enhance several pro-inflammatory cytokines, and consequently B cell stimulation and activation (Jacobson et al., 1996; Malnati et al., 2002; **Table 1**).

**Table 1:** Factors involved in HIV-1 and EBV interplay through B cell activation.

Factors		Mechanism(s)	Reference
HIV-1 proteins	Virions	Virions bind to B cells through CD21 and transmit infection to T cells	Moir et al. (2000)
	gp120	Binding to C-type lectins and induced immunoglobulin class switch recombination through a CD40-independent mechanism	He et al. (2006)
		Induction of TNF-α and hypergammaglobulinemia	Rieckmann et al. (1991)
	gp120 (X4 strains)	Increased proliferation capability of EBV^+^ B cells	Iyengar and Schwartz (2011)
	gp41	Induced activation of IL-6 and IL-10 pro-inflammatory cytokines	Takeshita et al. (1995)
	Nef	Activation of B cells and induction of hypergammaglobulinemia through ferritin produced via Nef-mediated activation of NF-κB	Swingler et al. (2008)
		Suppression of CD40-dependent immunoglobulin class switching	Qiao et al. (2006)
	Tat	Binding to Rb2/p130 and induction of cell cycle genes; modulation of cell cycle and increased proliferative capability of EBV^+^ B cells	Lazzi et al. (2002); Colombrino et al. (2004)
PAMPs^*^	LPS	Engagement and activation of TLR4; induction of pro-inflammatory cytokines	Brenchley et al. (2006)
	16S rDNA, CpG DNA	Engagement and activation of TLR9; suppression of EBV lytic cycle through inhibition of BZLF expression	Ladell et al. (2007); Zauner and Nadal (2012)
DAMPs^**^	mtDNA	Engagement of TLR9 and suppression of EBV lytic cycle	Zhang et al. (2010)
	HMGB1	Engagement of TLR2 and TLR4; induction of pro-inflammatory cytokines	Scaffidi et al. (2002)

* Pathogen-associated molecular patterns.

** Damage-associated molecular patterns.

It has been demonstrated that patients with high levels of EBV have higher levels of pro-inflammatory cytokines (IL-6, IL-10, TNF-α) and PAMPs (LPS) than patients with low EBV level, and that EBV load strongly correlates with the percentage of activated B cells (Petrara et al., 2012). In addition, patients under CD4-guided interruption of ART to reduce adverse drug effects show similar levels of HIV-1 plasmaviremia to ART-untreated patients and high levels of EBV (Petrara et al., 2012). The association between markers of B cell activation and the risk of NHL persists even after adjustment for CD4 T cell count, HIV-1 load and ART, thus supporting the independent role of these markers with NHL risk (Guiguet et al., 2009; Engels et al., 2010; Marks et al., 2013). These findings additionally support the concept that B cell activation, regardless of CD4 immune reconstitution, favors expansion of EBV-infected cells and the onset of EBV-related malignancies.

## CONCLUDING REMARKS

The risk of EBV-associated NHL in HIV-1 infected patients compared with the general population is mainly increased by impaired immunosurveillance against EBV and B cell chronic immune activation. The two mechanisms may contribute to the development of NHL subtypes in different ways. In HIV-1 infected subjects with low CD4 T cell count and severe immunedepression, a notable proportion of NHL is represented by PCNSL. These tumors are almost 100% EBV-positive, are less significantly linked to circulating levels of pro-inflammatory cytokines than systemic NHL (Breen et al., 2011), and represent the NHL subtype which most significantly declined after the introduction of ART. These observations are consistent with the concept that PCNSL primarily results from the loss of immunosurveillance against EBV.

**FIGURE 1:**
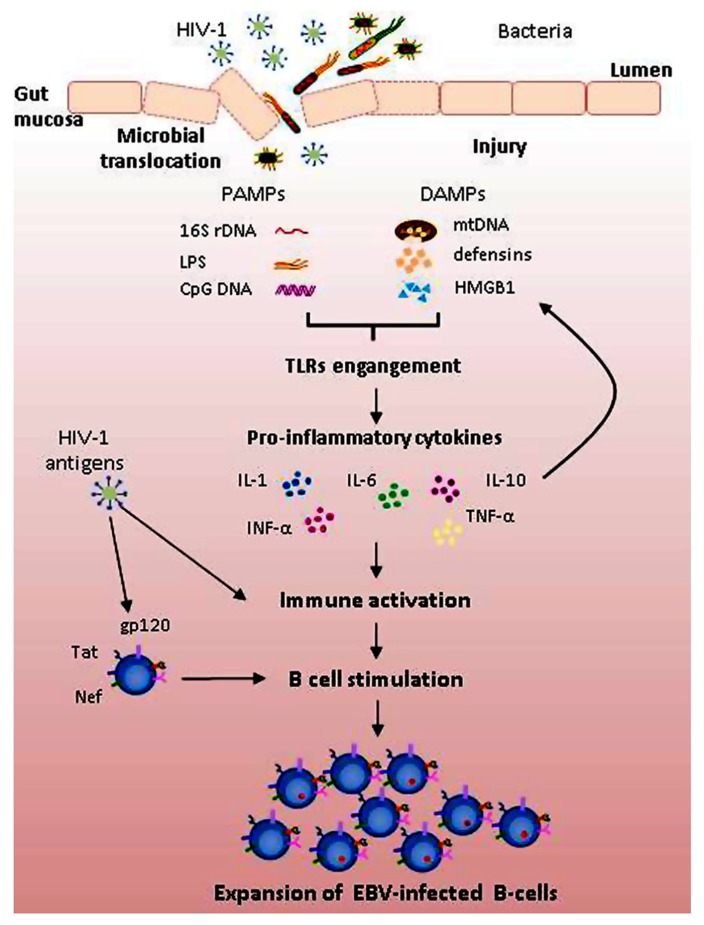
**Mechanisms potentially contributing to polyclonal B cell activation and expansion of EBV-infected B cells in HIV-1 infected patients.** HIV-1 acts as both a direct or indirect activator of B cells. Direct effects of HIV-1 include binding to B cells of HIV-1 viral proteins, such as gp120, Tat, and Nef, promoting polyclonal B cell activation. Indirect effects result from HIV-1-induced immune activation. Breakdown of gut mucosa induced by HIV-1 antigens increases intestinal permeability, resulting in translocation of microbial products. Pathogen-associated molecular patterns (PAMPs), such as LPS, 16S rDNA, and CpG DNA, and endogenous molecules created upon tissue injury (damage-associated molecular patterns, DAMPs), such as mitochondrial DNA (mtDNA), defensins and high mobility group box 1 (HMGB1) protein, by engaging Toll-like receptors (TLRs) activate signaling cascade leading to increased transcription of pro-inflammatory cytokines. In this context, both EBV-infected and uninfected B cells are activated and may undergo proliferation; EBV-infected cells may overcome uninfected ones due to higher replicative capacity and telomerase activation.

Systemic HL may occur relatively early in HIV-1 disease and the impact of ART in reducing these tumors is lower than that observed for PCNSL and other ADC. Chronic immune activation may considerably affect the onset of systemic NHL; chronic B cell activation, generated by HIV-1 antigens and the products of microbial translocations, may result in expansion of B cells and accumulation of genetic lesions over a prolonged period of time. According to this model, EBV does not represent a prerequisite for systemic lymphomagenesis; unlike PCNSL, systemic lymphomas may also be EBV-negative. Nonetheless, the oncogenic properties of EBV play a critical role; EBV-infected B cells may overcome EBV-negative B cells due to the proliferating activity and activation of telomerase (Terrin et al., 2007, 2008; Dolcetti and De Rossi, 2012); most systemic tumors are in fact EBV-positive (**Figure 1**). Current strategies to treat HIV-1 are efficient in decreasing HIV-1 plasmaviremia and restoring immune functions but may be not efficient enough in reducing immune activation (Regidor et al., 2011). The new strategies to treat HIV-1 should take into account this important parameter. The prevention of chronic immune activation may prevent the onset of EBV-related diseases.

## Conflict of Interest Statement

The authors declare that the research was conducted in the absence of any commercial or financial relationships that could be construed as a potential conflict of interest.
